# Towards accurate and reliable resolution of structural variants for clinical diagnosis

**DOI:** 10.1186/s13059-022-02636-8

**Published:** 2022-03-03

**Authors:** Zhichao Liu, Ruth Roberts, Timothy R. Mercer, Joshua Xu, Fritz J. Sedlazeck, Weida Tong

**Affiliations:** 1 National Center for Toxicological Research, U.S. Food and Drug Administration, Jefferson, AR 72079 USA; 2 ApconiX, BioHub at Alderley Park, Alderley Edge, SK10 4TG UK; 3 University of Birmingham, Edgbaston, Birmingham, B15 2TT UK; 4 Australian Institute for Bioengineering and Nanotechnology, University of Queensland, Brisbane, QLD Australia; 5 Garvan Institute of Medical Research, Sydney, NSW Australia; 6 St Vincent’s Clinical School, University of New South Wales, Sydney, NSW Australia; 7 Human Genome Sequencing Center, Baylor College of Medicine, One Baylor Plaza, Houston, TX 77030 USA

## Abstract

Structural variants (SVs) are a major source of human genetic diversity and have been associated with different diseases and phenotypes. The detection of SVs is difficult, and a diverse range of detection methods and data analysis protocols has been developed. This difficulty and diversity make the detection of SVs for clinical applications challenging and requires a framework to ensure accuracy and reproducibility. Here, we discuss current developments in the diagnosis of SVs and propose a roadmap for the accurate and reproducible detection of SVs that includes case studies provided from the FDA-led SEquencing Quality Control Phase II (SEQC-II) and other consortium efforts.

**Supplementary Information:**

The online version contains supplementary material available at 10.1186/s13059-022-02636-8.

## Introduction

Structural variants (SVs) are typically defined as genetic variants of greater than 50 base pairs (bp) in length and include insertions, deletions, duplications, and chromosome rearrangements [1]. Advances in sequencing technologies are constantly driving the improvements in SV identification, enhancing our understanding of the complex interplay between genetic makeup and associated phenotype [2]. Significant progress has been made in unraveling the importance of SVs in disease etiology, population genetic evolution, ethnic diversity, and gene expression regulation [3]. For example, the role of SVs in the key mutational process of various cancer types has been discovered including that rearrangements delete, amplify, or re-order large genomic segments [4–6]. Furthermore, SVs contribute to phenotypic diversity in neurological and rare diseases such as developmental disorders [7], autism spectrum disorders (ASDs) [8, 9], and schizophrenia [10]. Therefore, SVs have great potential in precision medicine via an understanding of SV patterns at the population level [11–15] and/or via implementation of pharmacogenomics (PGx) biomarkers [16].

Although great progress is being made in detecting SVs in the human genome and correlating this with phenotypic impact, accurately and precisely identifying SVs in specific samples and/or across samples is challenging [3]. The pressing challenges in SV calling are multifactorial, comprising SV types (insertion/deletion/re-order), size (where the allele often exceeds standard NGS technologies read length), genomics technologies (with differing abilities to identify variations in repetitive regions), and SV calling algorithms (which might use different thresholds and heuristics) [1, 3, 17].

SVs are responsible for more nucleotide changes than any other genetic variants in humans and other species. Many technologies have been developed to identify different types of SVs in the past 15 years, from cytogenetic-based detection (e.g., Karyotyping), array-based technologies (e.g., SNParray and FISH), short-read high throughput sequencing (e.g., NovaSeq), and linked-read sequencing (e.g., 10X Genomics Chromium Technology) to long-read sequencing (e.g., PacBio and Nanopore) [1, 18, 19]. Accordingly, numerous SV-calling algorithms have been developed specific to each technology [3]. For example, approximately 80 SV calling tools are available for short-read whole-genome sequencing (WGS) alone. Given these developments, how can we improve the detection of SVs with sufficient accuracy and precision for use in clinical diagnosis?

The accurate and reproducible detection of SVs underpins reliable clinical implementation and reduces the chance of misdiagnosis. Similar to the quality control steps made for SNVs and small indels [20, 21], initial steps have been made for SV detection [3, 22–26] with an effort to standardize (i) procedures to reduce false positives and false negatives through benchmark call set development (e.g., Genome in a Bottle (GIAB), Human Genome Structural Variation Consortium (HGSV)); (ii) high-confidence calling region establishment (e.g., GIAB), (iii) reproducible SV calling assessment, and (iv) reporting standards (e.g., GA4GH) and best practice guideline recommendations that are on-going, led by large consortiums and government agencies [22, 23, 27].

The Sequencing Quality Control Phase II (SEQC-II), led by the U.S. FDA, is the most current initiative to develop actionable best practice for sequencing data analysis. The aim is to define reproducible and accurate genetic variant calling to facilitate the development and regulation of precision medicine in clinical practice [28–32]. In this initiative, multi-platform and multi-lab sequencing data of the standard reference materials was carried out, encompassing the broad spectrum of wet lab factors (e.g., library preparation and gene capture strategy). The data generated is allowing the identification of key factors that impact SV detection, such as calling algorithms, SV size and type, and genomics technologies resulting in high-quality calling sets for further application.

**Fig. 1:**
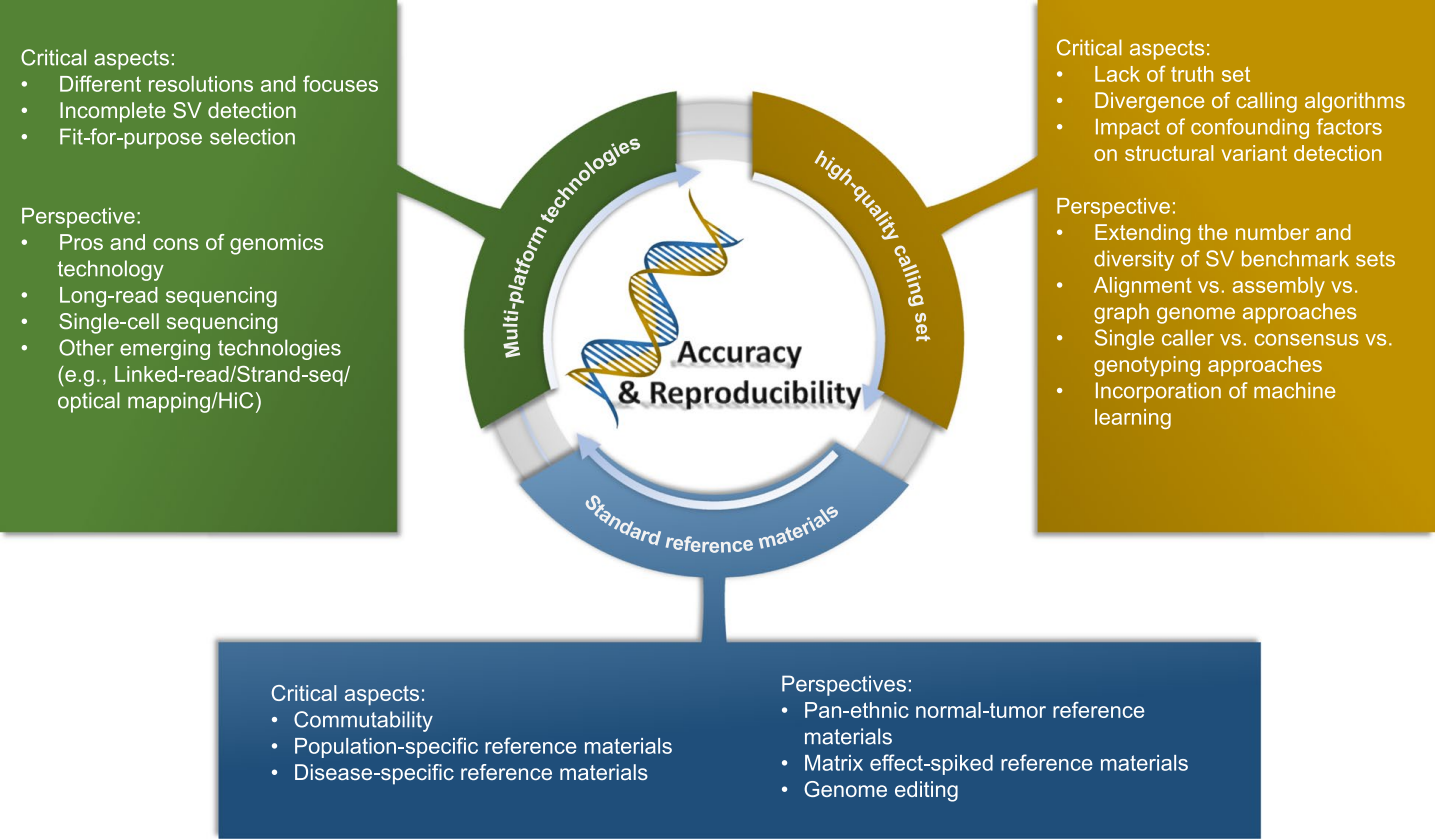
Roadmap towards accurate and reproducible SV detection

In this perspective, we discuss findings from these consortiums and identify gaps where current approaches for SV calling may be suboptimal, as well as propose potential solutions. Our analysis highlights three key components essential for an accurate and reproducible SV detection (Fig. 1): (1) characterization of standard reference materials, (2) determination of sequencing technology to improve SV detection, and (3) establishment of high-quality calling sets. We discuss the critical aspects of each component and propose potential solutions based on examples drawn from SEQC-II and other consortia. We also highlight the opportunity provided by artificial intelligence (AI) to potentially improve the accuracy of SV calling and propose specific deep learning solutions for the future.

### Characterization of standard reference materials

**Fig. 2:**
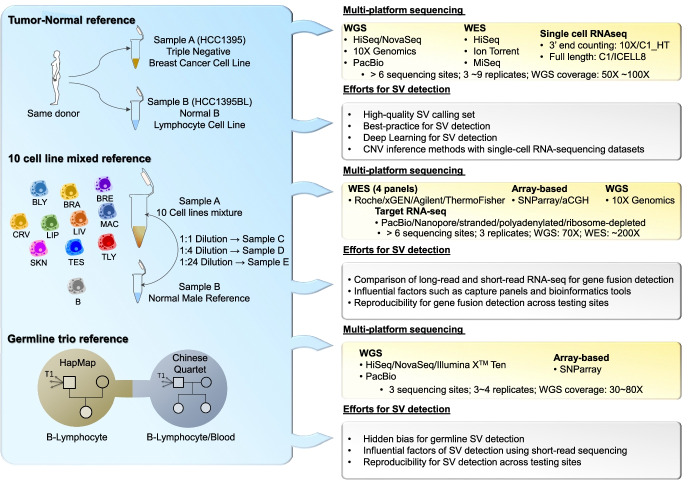
An insight into the reference samples and efforts for SV detection by the SEQC-II consortium. Great strides have been made in advancing SV detection by the SEQC-II consortium (Fig. 2). First, the SEQC-II consortium established high-quality SV calling sets based on multi-platform sequencing of tumor- normal reference samples and partially verified this using orthogonal methods, including PCR-based validation, cytogenetic array BioNano optical mapping, as well as fusion gene detected from RNA-seq [35]. Meanwhile, SEQC-II systematically evaluated the reproducibility of somatic SV detections across platforms and benchmarked the performance of various software tools. Leveraging the developed high-quality SV calling sets, they developed a deep learning-based calling algorithm for SV detection using the convolutional neural network (CNN). The proposed deep learning models achieved high robustness across multiple sequencing technologies for fresh and FFPE DNA input, varying tumor/normal purities, and different coverages, with significant superiority over conventional detection approaches in general, as well as in challenging situations such as low coverage, low variant allele frequency, DNA damage, and complex genomic regions. Furthermore, the CNV inference method was developed based on the generated single-cell RNAseq data. Second, the SEQC-II consortium comprehensively investigated the performance and confounding factors (i.e., long or short-read sequencing, capture panels, and bioinformatics pipelines) of gene fusion detection [28]. It was found that long-read sequencing achieved higher precision and discovered more novel fusion genes. Short-read sequencing achieved greater sensitivity for detecting known fusion genes correlated with the endogenous expression of targeted genes. Third, the SEQC-II consortium prioritized SV detection divergent sources by using multiple illumina-based short-read sequencing of the Chinese quartet reference samples. Interestingly, mapping methods are significant resources of calling variability, followed by sequencing centers and replicates. Surprisingly, SV supported by only one site or technical replicate often represented true positives defined by long-read PacBio sequencing, consistent with an overall higher false-negative rate for SV calling [36]

Reference standards can be used to measure the false-positive and false-negative rate of SV calling [33]. Specifically, a reference sample can be used to evaluate the accuracy and reproducibility of sequencing technologies, prioritize confounding factors that contribute to systematic errors, and establish best practice for SV detection. The use of a reference standard that includes well-characterized genetic material or synthetic spike-in control to calibrate sequencing platforms and measure the capability of SV detection is broadly supported [33, 34]. Well-characterized and broadly available reference samples are the foundation for assessing SV calling accuracy and reproducibility, as well as for understanding potential biases in data preparation or analysis. For quality control in the detection of SVs, reference materials need to be relevant for a wide range of SVs and variant allele frequencies (VAFs) to enable a comprehensive assessment [18]. The SEQC-II consortium established several working groups to characterize reference samples and then perform high-depth multi-platform sequencing across different laboratories to make progress in these areas, aiming to facilitate the qualification and validation of genetic variant detection and to promote reproducible science (Fig. 2).

#### Germline benchmarking

When reference materials are designed to improve assay development for inherited disease diagnosis, patient-parent trio samples offer more sensitive identification of the de novo SVs and any underlying predisposition mechanism. Examples of such trio-based reference materials (e.g., Reference Material 8392) were employed by the National Institute of Standards and Technology (NIST) [22] and the Human Genome Structural Variation Consortium [14] for the evaluation of SV detection.

Unlike these two working groups focusing on somatic mutations, the SEQC-II germline mutation working group focuses on a comprehensive and reproducible assessment of the detection of germline variants based on a trio sample set. For this, sample materials are the HapMap trio from the 1000 Genome project [37] and HG001 for the Genome in a Bottle (GIAB) led by the NIST [21]. Notably, Chinese Quartet reference DNA materials derived from normal B-lymphocyte cell line and blood samples are included, enabling the comparison of SV and SNV detection in different biological matrices and ethnicities [36, 38, 39]. Furthermore, the ABRF NGS phase 2 DNA-seq study leverages RMs (National Institute of Standards and Technology (NIST) RM 8392, known as the Ashkenazi trio; mother (HG004), father (HG003), and son (HG002), a family trio consented through the Personal Genome Project (PGP)) [40] to provide insight into currently popular sequencing instruments. Inter-laboratory and intra-laboratory DNA-seq replicates of the Ashkenazi trio are analyzed, as well as three individual bacterial strains and a metagenomic mixture of ten bacterial species to study the effects of GC content and library complexity [41]. These are novel and important studies since they not only assess like other studies the ability to call SV or SNV across certain data sets, but really dive into the question what causes the variability between different sequencing events. This is especially important when sequencing multiple samples (e.g., from a large cohorts) across different sequencing centers. All three studies revealed detail insights in the contribution of sequencing centers themselves including library preparations but further investigate the impact of different filtering (deduplication, QV recalibrations) and analytical approaches [36, 38, 39].

#### Somatic benchmarking

It is apparent that this is yet to be solved as many samples are “only” healthy cells derived from limited ethnic backgrounds. Especially in cancer, scientists are often interested in detangling complex SV that are chained, including virus insertions, somatic vs. germline mutations, low-frequency mutations escaping traditional diploid genomic patterns or even circularized DNA are not present in any of the reference materials yet. Several attempts have been made to develop reference material for somatic variants identification and NGS technology calibration in clinical laboratories. Specifically, the genome editing technologies such as CRISPR/Cas9 were adopted to biologically engineer mutation clones in mammalian cells [42, 43]. However, these reference materials could not be used to benchmark whole genome-based technologies and cover a broad spectrum of structural variant types. Additionally, Approaches to simulated sequence alignment data were also established to evaluate the variant calling algorithms for SNV and SVs [44]. Also, the two normal cell lines were titrated at various concentrations to mimic tumor heterogeneity. A normal cell line with engineered mutations or synthetic DNA spike-in is difficult, if not impossible, to engineer somatic mutations in a whole genome scale to mimic the complexity and heterogeneity of a cancer genome. Therefore, whether these reference materials could capture and represent patient tumor samples regarding VAF, inter- and intratumor heterogeneity, prevalent copy number alterations (CNAs), and complex chromosomal rearrangements are largely questionable about biased inferences and highlight the need for whole genome-based reference materials.

One approach could be to use tumor-normal or mixed cancer cell lines samples with different tumor purity and heterogenicity that may be closer to actual cancer patients. Cancer genomics aims to identify somatic (tumor-specific) variants with potential diagnostic, prognostic, and therapeutic implications and to detect germline variants with inherent information for both patients and their families. Although substantial clinical tumor testing does not currently involve analysis of a matched germline sample due to cost and time to delivery, concerns have been raised that the detected genetic variants are truly somatic variants [45]. Moreover, consortiums such as NHGRI/NCI Clinical Sequencing Exploratory Research Consortium Tumor Working Group and the American College of Medical Genetics and Genomics (ACMG) released a set of guidelines recommending that laboratories performing cancer sequencing tests should include germline variants [46, 47]. The International Cancer Genome Consortium used tumor-normal sample pairs from two different types of cancer, chronic lymphocytic leukemia (CLL), and medulloblastoma (MB), for a comprehensive assessment of somatic variants identification, emphasizing on the imperativeness of real, not simulated, mutations are more helpful in dissecting performance of mutation callers on establishing whole-genome somatic variant signatures incredibly complex SVs [48]. However, the ratio of DNA amount between tumor and matched control in the reference materials developed by ICGC is predefined with low mutation burden and minimal structural changes, limiting its utility to assess the detection limit of various genomics technologies.

Specifically, the SEQC-II somatic mutation working group developed a tumor-normal reference sample using a normal B-lymphocyte cell line (i.e., HCC1395BL) and a triple-negative breast cancer cell line (i.e., HCC1395) from the same donor purchasable through the American Type Culture Collection (ATCC) [35]. The HCC1395 cell line has been characterized with conventional genomics approaches such as cytogenetic analysis [49] and array-based comparative genomic hybridization [50], consisting of rich genetic variant types including ~40,000 SNVs, ~2000 small indels, CNAs covering over 50% of the genome, more than 250 complex genomic rearrangements [51], and an aneuploid genome and BRCAness [52]. Moreover, the HCC1395 DNA was pooled with HCC1395BL DNA at different ratios to create a range of admixtures that mimicked tumor purity levels of 100%, 75%, 50%, 20%, 10%, 5%, and 0%. Furthermore, this tumor-normal reference sample set may also mimic different biospecimen types (i.e., fresh vs. formalin-fixed, paraffin-embedded) for investing the effect of fixatives and sample handling on variant detection.

While large whole-exome and whole-genome sequencing studies are capable of providing new insights of genetic variants into cancers, clinical adoption of these approaches is still lagged and is not routinely offered by clinical laboratories [53]. In contrast, large, low-cost, and short turnaround time targeted cancer panels have been widely utilized in clinical laboratories. Encouragingly, some panel-based NGS have been approved or cleared by regulatory agencies such as U.S. FDA to promote precision medicine (https://www.fda.gov/medical-devices/in-vitro-diagnostics/list-cleared-or-approved-companion-diagnostic-devices-in-vitro-and-imaging-tools). To enhance the clinical application of panel-based sequencing for cancer diagnosis, we suggest it is indispensable to establish reliable, robust, continuous, and generally available genomics reference samples for assessing and calibrating different NGS assays.

VAF of somatic mutation (e.g., VAF < 20%) is far less than the VAF in germline cells (e.g., ~50% and ~100%). It is technically challenging to utilize normal cell lines to establish a reference material covering a wide range of VAF magnitudes. Commonly available commercial reference samples typically consist of the limited number of genes and variants with distinct VAF ranges, hampering its utility for benchmark NGS assays in genetic variants with lower VAF (< 2~5%) and resulting in the overall variant detection performance is inversely correlated to the VAF of the analytes targeted (https://www.horizondiscovery.com/reference-standards/type/oncospan). To overcome the shortcoming, the SEQC-II Oncopanel working group synthesized sample A by equal mass pooling gDNA of ten cancer cell lines that were used to make the Universal Human Reference RNA (UHRR) (Catalog #740000, Agilent Technologies) to ensure the wide coverage of actionable cancer genes under different VAF magnitudes [28]. Sample A permitted the investigation of 40,000 variants down to 1% allele frequency with more than 25,000 variants having less than 20% allele frequency with 1653 variants in COSMIC-related genes, which is 5~100× more than existing commercially available samples. Also, a cell line derived from a normal male individual (Agilent OneSeq Human Reference DNA, PN 5190–8848) (termed “Sample B”) was characterized and employed as a negative control for somatic variants but also generating different genomic backgrounds. By titrating sample A into a control sample B with varying ratios of mixing (i.e., 1:1/4/24/124), samples C, D, E, and F were created to mimic the somatic mutation noted in extremely low VAF (as low as 0.02%), a range suitable for assessing liquid biopsy panels for detecting mutations in circulating tumor DNA [54].

Commutability, defined as the ability of reference materials to perform comparably to actual patient samples, is one of the most critical parameters in qualifying reference materials [33]. To establish a more objective quality assessment of SV detection, we suggest developing additional biological certified reference materials as follows:

1. Establish pan-ethnic normal-tumor reference materials. The ethnic diversity of SVs regarding size and types across different genome regions (e.g., rich GC contents or low complexity) in inherited diseases and cancers has been widely reported [23, 55]. Trio-based reference materials from different ethnic backgrounds have been developed, such as trio samples of Han Chinese, Puerto Rican, and Yoruban Nigerian ancestries developed by the Human Genome Structural Variation Consortium [14] and the Ashkenazi family trio (HG002, HG003, and HG004) in the GIAB [22]. However, few pan-ethnic normal-tumor reference materials are publicly available and/or purchasable. Therefore, an effort for coordinated, local implementation of the development of normal-tumor reference materials from diverse ancestries or admixtures is highly recommended to improve our understating of somatic SV differences in local populations.
2. Matrix effect-spiked reference materials. The SV detection limit within formalin-fixed, paraffin-embedded, or liquid biopsy-based samples from patients is much lower than that in normal tissues. Furthermore, some complex effect of fixatives such as in vitro fertilization (IVF) and preimplantation genetic diagnosis (PGD) screening help patients to select embryos free of rare diseases [34]. As the part of efforts from the SEQC-II consortium, we proposed synthetic internal standards (IS) and methods for better control for technical error in NGS in assessment of circulating tumor DNA specimens, enabling measurement of low AF mutation not detected by current practices. The proposed Synthetic spike-in IS could be effective way to mimic the complex effect of fixatives and evaluate the NGS testing [56].
3. Use genome editing for spiked specific SV types in the reference materials. Genome engineering technology with programmable nucleases (e.g., ZFNs, TALENs, and CRISPR/Cas9) has been well-established, enabling precise and efficient genome-editing spiking of specific SV types into cells, and offering the opportunity to develop synthetic reference materials [57]. For example, CRISPR/Cas9-based genome-editing protocols have been developed for the direct generation of deletions, duplications, and inversions of up to one million base pairs in zygotes [58]. However, a close examination of the potential unexpected off-target variants in the engineered cell line should be considered [59].

### Determination of sequencing technologies to improve SV detection

Advances in genomics technologies continually improve the resolution of SV detections [1, 18]. From the use of microscopy to visualize karyotypes of short, condensed chromosomes to long-read sequencing to identify complex rearrangements that consist of multiple combinations of SV events, Table 1 summarizes some representative NGS technologies used for SV detection. The ability to more sensitively detect SVs and resolve more complex rearrangements has resulted in an exponential increase in the number and research on SVs identified over the past 15 years. However, the genome’s intrinsic complexity, the technical errors introduced during sample preparation, and the limitation of the existing sequencing technologies have left a substantial fraction of SV undetectable and much of their complexity remains hidden.

**Table 1:** A comparison among different sequencing technologies for structural variant detection

Platform	Read length	Cost	Comments	Run time
Short reads (Illumina)	NovaSeq: up to 250 bp	$	Short-read NGS performs well for >1kb regions. It struggles with shorter CNV detection 50-500bp, and in complex genome regions	NovaSeq: 0.15Tb/day
10X Genomics Chromium	Up to ~100 kb	$$	Sparse sequencing rather than true long reads; more complicated to align, with poorer resolution of locally repetitive sequences. However, 10X Genomics Chromium is currently discontinued	-
PacBio SMRT sequencing	10–15 kb (average) and up to 100 kb	$$$	HiFi: long reads (10-20kbp) of high fidelity having a similar error rate as Illumina. CLR: Longer raw reads have high error rates dominated by false insertions; requires new alignment and error correction algorithms	20 Gb/day
Oxford Nanopore	averaging ~10 kb and up to 2 Mb	$$$	Raw reads have ~5% error rates dominated by false deletions and homopolymer errors; often requires new alignment and error correction algorithms	A MinION Flow Cell : ~ 25 Gb/day
Hi-C-based analysis	<100 bp	$$	Sparse sequencing with highly variable genomic distance between pairs (1 kb to 1 Mb or longer); Detection may result from random chromosomal collisions Less than 1% of DNA fragments actually yield ligation products. Due to multiple steps, the method requires large amounts of starting material	Whole analysis within 28 hours
BioNano Genomics optical mapping	~250kb or longer	$	Limited algorithms to discover high-confidence alignment between an optical map and a sequence assembly	100x coverage of 3 human genomes is collected in less than 6 hours

An increasing body of literature has demonstrated the benefits of employing multi-platform genomics technologies for more comprehensive SV detection across the human genome [14]. Chaisson et al. [14] used a suite of long-read, short-read, strand-specific sequencing technologies, coupled with optimal mapping and SV calling algorithms to provide a complete spectrum of haplotype-resolved SVs in human genomes from three sample trios. Specifically, their multi-platform strategy detected three- to seven-fold more SVs than most standard high-throughput sequencing studies. Moreover, more inversions located within critical genome regions were discovered, associated with rare recurrent microdeletions and microduplication syndromes [60]. Despite these advantages, the use of multiple sequencing technologies to resolve SVs may be impractical. Furthermore, are there certain biases introduced when sequencing the same sample multiple times?

The SEQC-II adopted multi-platform and multi-lab designs for a comprehensive assessment of reproducibility and accuracy of the detection of SVs. The SV working group set out to investigate the reproducibility and variability of SV calls when the sample was sequenced across multiple sequencing instruments or in different laboratories [31, 32]. Interestingly, current approaches produced a level of variability often associated with false negatives (i.e., missed SVs) in SV calls with current methodologies. The somatic mutation working group used the developed tumor-normal materials coupled with multi-platform sequencing technologies, including short/long/linked-read sequencing and high-throughput chromosome conformation capture (HiC). Moreover, they also performed multi-platform, single-cell RNA sequencing technologies to establish best practice for single-cell RNAseq analysis [61]. The Oncopanel working group employed four commercialized WES panels with multiple library preparations and 10X Genomics linked-read sequencing for the individual cell lines to generated high-coverage sequence data on the developed reference samples. SVs reported by linked-read sequencing data were then compared with gene fusion events detected in RNA sequencing data of UHRR. Meanwhile, the linked-based WGS and array-based SNParray/aCGH data were also generated for investigational and confirmation purposes. The germline working group utilized most Illumina-based short-read sequencings such as HiSeq 2000, NovaSeq to XTen, and the Chinese Quartet were also sequenced using long-read PacBio.

Although the promise of multi-platform genomics technologies for complete SV detection is apparent, it may be more appropriate to use a combination of different sequencing technologies; insight into their different strengths and weaknesses is key for effective deployment. The pros and cons of different genomics technology for SV detection were discussed extensively elsewhere [1, 17, 62]. Here, we focus on key points in optimizing the selection of multi-platform technologies for enhancing the accuracy and comprehensiveness of SV detection.

First, a multi-technology approach is not scalable given the costs and DNA, cell, or sample requirements such as quantity and quality for the clinical samples. One technology that may enable more accurate SV detection is known as long reads [3, 63]. Despite the fact that long reads may have a higher error rate (HiFi: ~0.1–1%, ONT: 3–8%), they have shown remarkable performance and resolution of SVs across the genome. In contrast to short reads, they often identify almost twice as many SVs, many of which include novel sequences (i.e., insertions). Furthermore, long-read sequencing significantly reduces the overall false discovery rate seen with short reads (e.g., translocations that shadow repeat expansions [63]). However, long-read sequencing remains costly, and the DNA requirements (quality and quantity) are not always achievable. Furthermore, the HGSV and others have also demonstrated the limitations of long reads, such as accessing and recovering certain complex regions of the genome (e.g., centromeres or telomers) or other complex SV types where more targeted essays might perform better (e.g., StrandSeq [64]).

Second, given the trend of reducing sequencing costs and improving yield as well as error rate, multiple long-read sequencing projects are underway from the assembly of individual genomes through the study of the genomic architecture of individual human cells [65]. This trend is likely to continue. However, will it replace short-read sequencing? The answer is a tentative no, given the scalability, the DNA requirements, and the costs.

There are multiple short read-based assays currently available. Linked reads constitute a fast and inexpensive method that provided information across a long molecule (e.g., 100kbp), leading to improvements in mappability and phasing. In theory, this approach could improve SV calling itself, but the software for achieving this was limited. Other interesting concepts such as HiC and StrandSeq also show promises. StrandSeq enables the accurate detection of inversions but requires laborious preparation, and there is not yet a standardized kit available. However, both technologies are limited by the mapping into repeats given their read length of 100–150 paired-end reads. Nevertheless, a combination of StrandSeq and PCR-free WGS may improve the detection of SVs at a cost and sample requirement that outcompetes long reads.

Currently, WGS remains the workhorse of genomics, with million genomes sequenced every year. Therefore, we need to establish robust pipelines, ensure technical reproducibility, and understand the risk of bias. SEQC-II has so far approached this by sequencing samples across multiple centers to improve our understanding of potential variabilities and the impact on SV detection [66].

### Establishment of high-quality SV call sets

The evolution of genomics technologies has also resulted in the proliferation of different SV calling algorithms. Over 85 publicly available SV calling algorithms have been developed for different NGS data types [26]. These algorithms aim to identify the divergence between the reference genome and the sample reads, which examine the following read features or combinations: read-pair, read-depth, split-read, and de novo or local assembly [1]. The motivation behind continually producing new SV calling algorithms is to improve on previous shortcomings, resulting in better precision and recall rate, speed and user-friendliness, and handling specific SV types. To establish newly developed SV calling algorithms, researchers compared outcome with previous calling algorithms using simulated and actual NGS data and highlighted their superiority from a particular perspective. Consequently, downstream users have little to guide them when choosing the best “fit-for-purpose” algorithms since they find every algorithm claims to be the right option. As a result, community efforts comparing the SV calling algorithms are being carried out to determine relative advantages and disadvantages and suggest best-practice for SV algorithm selection [25, 26].

This work started over a decade ago. SV calling is improving but has by no means reached the reliability and quality of SNV calling. While multiple reasons may attribute to this problem, SV alleles are longer and often more complex than SNV which can be contained in a single-short read. Nevertheless, what steps can be taken to improve the field further or spark a new evolution of methodologies and approaches? Here we highlight some potential directions and discuss their promises. These are (1) extending the number and diversity of SV benchmark sets, (2) alignment vs. assembly vs. graph genome approaches, (3) single caller vs. consensus vs. genotyping approaches to improve SV precision, and (4) incorporation of machine learning and AI.

#### Extending the number and diversity of SV benchmark sets

Over the past few years, multiple methods have been proposed and implemented to simulate SV (e.g., VarSim [67], SURVIVOR [68], etc.) and simulate read data (e.g., NanoSim [69], PBsim [70], etc.) [71]. While these methods are helpful for rapid, early understanding of the utility of an SV caller, they often under- represent the complexity of SVs either at the level of the allele itself or in the regions they tend to occur (e.g., repetitive). Therefore, they are no replacement as yet for high-quality benchmark sets such as HGSV or GIAB, comprising by now 10–20 benchmark sets across multiple individuals and ethnicities. Nevertheless, these SV benchmark sets range in quality and accessibility and thus also in their ability to be leveraged for benchmarking SV callers. For example, for SV, NA12878 from GIAB is an older and lower quality benchmark set compared with the HG002-4 trio, mainly because NA12878 has multiple redundant SV calls and imprecisions. HG002-4, however, is of high-quality spanning over 90% of the human genome, but is only available for hg19 at the moment [22]. HGSV also produced high-quality assemblies, leveraging multiple technologies over the past years. Having the latest releases will improve the phasing, continuity, and accuracy of their assemblies and thus also the SV calls themselves. These samples are derived from different genders and a few different ethnicities, but so far do not cover all ethnicities and are missing disease phenotypic benchmark genomes. At the time of writing the review, no one from our knowledge have done a head-to-head comparison of the GIAB and HGSV released call set also potentially because both seems to be of very high-quality given all the validation and QC steps done. Both efforts focus mainly on insertion and deletions (especially GIAB) as they represent the majority of SV. HGSV also included inversions, but both are not addressing the biggest issue around translocations or other more complex SV types [72]. Short read SV call sets often report in the access of up to 4000 translocations of which ~50–70% are repeat extensions [63]. In contrast most methods can detect 50–300bp insertions these days (e.g., delly and manta), but fail at larger size ranges. The high-quality calling set generated from SEQC-II tumor-reference samples is potentially the first step in the right direction (Fig. 2). Here, we summarized the data generated from SEQC-II consortium efforts for trigger the community’s interest to further explore the potential improvement for SV detection (Table 2).

**Table 2:** Benchmark datasets generated from SEQC-II consortium efforts and potential application in SV detection

Working group	Reference samples	Benchmark data	Potential benefit for SV detection	Link
Somatic mutation [30, 32, 66, 73–76]	**Tumor-normal** sample: HCC1395BL as normal and HCC1395 as tumor	*Fresh DNA*: **WGS** - HiSeq, NovaSeq, 10X Genomics, and PacBio) **WES** - Hiseq and Ion Torrent **AmpliSeq** - MiSeq **Microarray** -AffyChip CytoScan HD	• Somatic SV benchmark establishment • Low allelic frequency (LOF) somatic SV detection in liquid biopsy or FFPE samples • Deep learning-based somatic SV detection • Reproducibility and repeatability assessment of somatic SV detection based on multiple sample and design	**All raw data** (FASTQ files): NCBI’s SRA database (SRP162370) **VCF and source code**: ftp://ftp-trace.ncbi.nlm.nih.gov/seqc/ftp/release/Somatic_Mutation_WG/ **BAM files**: Seven Bridges’ s Cancer Genomics Cloud (CGC) platform and license is needed.
		*FFPE/mixed DNA*: **WGS/WES**: Hiseq		
		*Fresh cells*: **scCNV**: 10X Genomics		**scCNV data**: SRA repository under accession code no. PRJNA504037. **Source code**: https://github.com/oxwang/fda_scRNA-seq and https://codeocean.com/capsule/0497386 or 10.24433/CO.1559060.v1.
Oncopanel [28, 29, 54, 56, 77]	**Sample A**: ten cancer cell line mixture **Sample B**: a normal male cell line (Agilent OneSeq Human Reference DNA, PN 5190–8848) **Spike in samples:** 5% AcroMetrix spikes-ins + Sample B	*8 pan-cancer gene panels*: **WES**: HiSeq, NovaSeq, Ion Torrent, Nanopore, Stranded RNAseq **WGS**: 10X Genomics **Microarrays**: SNP array and aCGH	• Reproducibility and repeatability assessment of actionable somatic SV assessment • Benefit of gene fusion detection by integrating DNAseq and RNAseq	**FASTQ or BAM**: BioProject PRJNA677997 - https://www.ncbi.nlm.nih.gov/bioproject/PRJNA677997. **VCF/BED**: https://figshare.com/projects/SEQC2_Onco-panel_Sequencing_Working_Group_-_PanCancer_panel_Study/94520
Germline mutation [36, 38, 39]	Chinese Quartet samples (B-lymphocyte cell line and blood samples)	**WGS**: Hiseq, NovaSeq, illumina X10, PacBio **Microarrays**: SNP array	• Influential factors on reproducibility assessment for germline SV detection • Germline SV detection concordance between B-lymphocyte cell line and blood samples • Deep learning-based somatic SV detection • Cross check the best practice of germline SV detection with NIST efforts	**Raw data**: BioProject PRJNA723125 (HapMap samples) https://www.ncbi.nlm.nih.gov/bioproject/PRJNA723125/ and NODE OEP001896 (Chinese Quartet Samples) https://www.biosino.org/node/project/detail/OEP001896 **Source code**: https://github.com/justwalking2017/SEQC_WG3_Script
	HapMap samples (HG001)			
	HapMAP Ashkenazi Trio	**WGS**: HiSeq, BGISEQ, MGISEQ, NovaSeq **WES**: Ion proton and Ion S5		**Raw data**: BioProject PRJNA646948 (https://www.ncbi.nlm.nih.gov/bioproject/?term=PRJNA646948), within accessions SRR12898279–SRR12898354 **Source code:**https://www.github.com/jfoox/abrfngs2
	Bacterial genomes (ATCC MSA-3001)	Miseq, Ion PGM, Ion S5, MinION, Flongle, and GenapSys		

Furthermore, some cancer cell lines (e.g., SKBR3) have already been heavily sequenced and studied, but no official benchmark SV set is available. Cancerous but also other human disease genomes will be important as these include challenging regions (e.g., oncogene amplification) that are not trivial to resolve. Additionally, the need to include different ethnicities and the potential for novel sequences (e.g., in African Americans) suggests that there is still some way to go. In any case, the current existing benchmark sets are already showing an improvement in SV calling methods and methodologies and are also providing standards for wet lab technology development.

#### Alignment vs. assembly vs. graph genome approaches

There is often a debate about the best approach to identify SVs, either at scale (multiple hundred to thousand samples) or comprehensively (complex structures within a sample). Mapping (i.e., the alignment of reads to an established reference genome) compared to de novo assembly (i.e., the reconstruction of the sample genome without any template) often has different advantages and disadvantages [3]. In general, de novo assemblies require more coverage to provide a continuous reconstructed sequence without gaps or uncertainties, while mapping strategies rely on the completeness of the reference genome [3, 17]. While these two particular criteria might sound trivial, they present a challenge with multiple implications for the performance of different approaches. For example, mapping an African sample to the human genome (GRCH38) may result in some unaligned reads due to the absence of reference sequences in the human reference genome. SV caller tries to identify this sequence later on as insertions are often missing (e.g., short reads) or at least missing to reconstruct larger insertions (multiple kbp for long reads). Thus, mapping approaches often succeed in scaling and requiring less expensive sequencing technologies or coverages to succeed. Their disadvantages, however, are often that more complex alleles are hard to resolve and the reconstruction of larger (multiple kbp) novel sequences often fails [3].

Alternatively, for assemblies, there are still risks of over-merging or splitting regions due to high (e.g., immune regions) or low (e.g., LOH) heterogeneity. This is mainly due to the central paradigm of an assembly of when are two genomic regions the same and some sequencing artifact altered them slightly or if these are indeed two different regions. Prominent assembler for short reads (e.g., SPAdes [78]) or even long reads (e.g., Canu [79], hiFiasm [80], Shasta [81]). Furthermore, the current bottleneck is often caused by the accuracy of genomic alignment [82]. Still, de novo assembly is currently the way to establish new benchmark SV sets [83]. The suggestion that de novo assembly will replace mapping for large-scale genomics is currently not foreseen.

Another option that might be able to combine these advantages over time is a graph genome approach. Here multiple genomes (e.g., representing different ethnicities, cancer samples, or other groups) are first assembled into high-quality genomes and then utilized to improve mapping and thus variant detection. As one can imagine, if this graph genome carries a novel sequence or complex SV, it enables the detection of these in the analysis of other samples. However, there are apparent limitations such that there needs to be a balance between comprehensiveness (i.e., including every private allele) and complexity (too many alternative SNVs and SVs making the graph ambiguous). While these problems are being addressed, graph genomes have already shown significant promises for SV calling: Paragraph [84] and VGTool [85]. As such graph genomes are still seen as a novelty and not many robust and established methods exist. Nevertheless, this will likely change in the near future with ever more high-quality assemblies being made available [82].

#### Single caller vs. consensus vs. genotyping approaches to improve SV precision

The SV detection limit differs across different types (e.g., deletions vs. insertions), sizes (e.g., 50 bp vs. 50 kb), and genomic regions (e.g., repeats). SV calling algorithms were developed based on different heuristics, hypotheses, and thresholds, resulting in a considerable divergence. This is illustrated by comprehensive benchmark papers across SV callers [25, 26]. Both authors found no single SV calling algorithms could perform at the top across different SV types and sizes. The high divergence on the precision and recall rate between the simulated data and actual data highlights the low commutability of simulated data. Long run time and increased memory requirement do not guarantee better SV detection performance. Importantly, the assembly-incorporating callers such as GRIDSS [86] and Manta [87] outperformed other callers with the actual data, consistent with the conclusion reached by Kosugi et al. [25]. We suggest further investigating whether SV caller performance findings drawn from these studies could be extrapolated to the different sequence depth and tumor purity through SEQC-II multi-platform and multi-lab sequencing data. One aspect to consider is of course also the size of the variants that are of interest. For example, very large alterations of multiple Mbp or even chromosome arms are often better detected by coverage approaches than SV callers (i.e., utilizing alignment signals) themselves [3]. That is often the case since these large CNV events do not have resolved breakpoints. Thus, a CNV approach over coverage or B-allelic (i.e., using SNP called) often performs better to characterize these.

The use of consensus calls generated from multiple SV callers can achieve better precision and recall rate [25, 26]. Nevertheless, unions of all SVs calls across multiple SV calling methods might result in a high false-positive rates than a single caller has despite often having a higher sensitivity [88, 89]. This is as the new SV set will incorporate also the falsely identified SV calls from the individual SV callers. On the other hand, a too restrictive approach to require, for example, three or more SV caller to agree leads often to a low sensitivity but high precision [88, 89]. Thus, the balance is often hard to achieve. Meta-callers, created by combining multiple SV calling algorithms have been proposed [88, 90, 91]. The proposed meta-callers could be mainly divided into rule-based strategies and machine learning with discriminating features based on the adopted ensemble strategy. However, these meta-callers either focus on a few SV-calling algorithms or need a “true set” to train the optimized model, leaving room for further improvement. The primary problem with rule-based strategies is the absence of unified criteria to prioritize SV calling results from different callers. For example, a few meta-callers such as Parliament2 [91] and SVMerge [92] focus on coordinate overlap through soft clip comparison and adjustment with local assembly. Parliament2 incorporates the usage of five pre-defined and optimized SV calling methods and allows users to decide on an optimized SV caller combination through quality score determined by SURVIVOR [68] and genotyping with SpeedSeq [93]. Alternatively, MetaSV adopts a priority-based combination strategy, exemplifying the weight of read pair-based callers over the split read-based ones. For the employed ensemble strategy, a true set was utilized for training SV callers either through machine learning strategies (e.g., CN-Learn [94]) or statistical measurements for each different SV type and size condition (e.g., Parliament2 [91] and FusorSV [88]). All three methods (Parliament2, FusorSV, and MetaSV) either incorporate or require a certain set of SV calling methods over short reads to improve their runtime and consensus performance. Noteworthy is that SURVIVOR offers a generalizable framework; however, this might need to be optimized further for a comparison across different technologies or methodologies (e.g., assembly vs. mapping-based SV calls) as the representation of SV are often different. Thus far, SURVIVOR has been used across different sequencing technologies successfully [95].

Another less-studied area is the use of a SV genotyper after initial SV calling. Here, a SV genotyper uses a VCF with predefined SVs and summarizes the evidence for these SVs across a given sample or bam file. There are currently multiple SV genotyping methods available such as STIX, Paragraph, SVTyper, GraphTyper, and others. Their performance varies similar to SV callers over sizes and types of SV [89]. Still, one potential benefit is that SV genotypers have, in general, fewer constrains and thresholds to identify SV as they operate on a given list of SVs. Thus, this might be able to improve recall while retaining high precision compared to other consensus methods that rely only on SV discovery methods.

#### Incorporation of machine learning and AI

Machine learning algorithms especially neural networks have impacted almost every facet of our daily life and have revolutionized genomics [96–99]. For example, deep learning has shown merits in enhancing SNV/small indel detection, outperforming most well-established SNV callers [100]. Inspired by DeepVariant and others, we believe that machine learning has a great potential to improve every step of the proposed roadmap towards accurate and reproducible SV detection.

The fundamental difference between conventional SV calling algorithms and deep learning approaches lies in retrieving the SV information from the BAM file. Traditional methods aim to detect the divergence between the sample sequence and reference at a per read level first. In contrast, deep learning approaches such as convolutional neural networks (CNNs) transform variant detection as a classification problem by considering the BAM file or its genomic regions as an image [100].

**Fig. 3:**
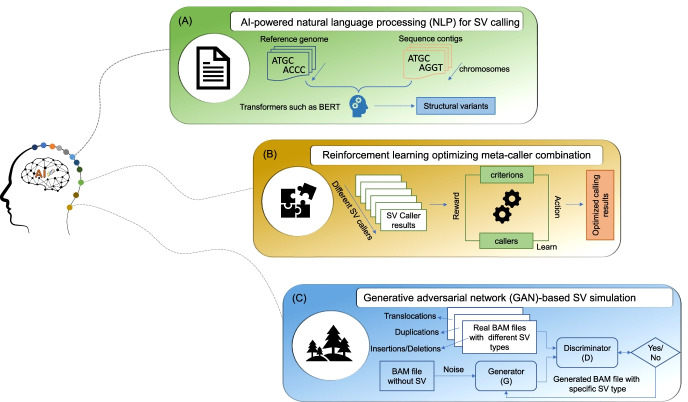
The role of AI in promoting SV detection. AI-powered natural language processing (NLP) for SV calling. Considering the suboptimal performance of different SV calling algorithms concerning completeness and accuracy, we suggest that deep learning may be an alternative worth further exploration. CNNs are the primary deep learning algorithm investigated for SV detection, which considers the BAM files as image. Rapid development of deep learning algorithms such as AI-powered NLP not only provided unprecedented innovation for information retrieve from free-text documents but repositioned in other type of biological information such as chemical structures and protein sequences [104]. Here, we developed a hypothesis by resembling chromosomes as paragraphs, the sequence reads as sentences, and different A, T, G, C combinations (e.g., tandem repeats and microsatellite) as vocabularies. Subsequently, the AI-powered language models such as different transformers [105–107] could be utilized to digest genome sequence as human beings read a book. The difference between the sample genome and reference genome (i.e., variants) could be extracted by compared transformer-based genome embedding, which is very similar to the rationale behind de novo assembly (**A**). Reinforcement learning optimizing meta-caller combination. There is the potential to integrate multiple callers using more sophisticated approaches than simple heuristic union/intersection rules for improving SV detection. Artificial intelligence (AI) may be a solution. The rapid evolution of emerging genomics technologies suggests that improved SV detection should be taking place. The ideal combination strategy for combining different SV callers is to take advantage of each SV caller and eliminate the false positives, which fits well the concept of reinforcement learning. Reinforcement learning is a branch of deep learning that focuses on how intelligent agents ought to take actions in an environment to maximize cumulative reward [108]. AlphaGo is an excellent example of reinforcement learning applications [109]. Reinforcement learning could be utilized to develop the intelligent ensemble SV callers to maximize SV detection performance (**B**). For each type of SV, the combination for each SV caller could be learned by minimizing the loss function that measures the divergence between called SV and ground truth. Ultimately, reinforcement learning-based ensemble SV callers allow the integration of any individual caller, incorporate different SV types, and incorporate the advantages of newly developed technologies. Generative adversarial network (GAN)-based SV simulation. A true set is the key to investigate the accuracy and reproducibility of SV detection. Unfortunately, the complexity of the SV events and associated genome properties are central to the whole picture of SV events in the sample, hampering objective evaluation. Many reports suggest that simulated ground truth cannot recapitulate genome and SV characteristics and mimic the actual patient situation. Therefore, the simulated SV truth sets with high commutability are urgently needed. The generative adversarial network (GAN) is a deep neural network framework integrating a generative model and discriminative model to generate new data similar to the statistical distribution of the training set [110]. GANs have been widely applied in image generation in fashion, art, and advertising and have attracted much attention in the scientific community. For example, one type of GAN model, named DeepFake, has been utilized to predict cell type-specific transcriptional states induced by drug treatment [111]. Here, we envision a generative adversarial network (GAN) to simulate the binary alignment map (BAM) file spiked in different SV types based on the actual data (**C**). The proposed GAN model collects the high-quality BAM files with varying SV and length types from the real data, such as SEQC-II and other consortium efforts, as a training set to generate the target SV spiked BAM file. The potential benefit of the proposed GAN model is the simulated SV spiked BAM file could maximize the preservation of the original matrix effect of real data such as VAF level and tumor purity

A few initial attempts to apply deep learning algorithms for SV detection have been conducted, and some encouraging results were obtained [101–103]. One example is DeepSV, which utilized the CNN model for training the visualized sequence in the 1000 Genomes Project for deletion/detection and yielded better accuracy than the conventional SV callers [102]. The proposed DeepSV could be extended to detect other SV types using the SEQC-II high-quality calling set and NIST germline SV calling set [22]. Another way to utilize the power of machine learning is for the assessment of the quality or accuracy of an SV. It is interesting to note that multiple visualization methods around SV enabled a fast and reliable filtering of SV as a replacement for manual assessments. However traditional SV callers lack this intuition of the human eye or mind. Samplot-ML is a method that combined the visualization of SV and was trained on false calls from SV methods to better distinguish true from false SV candidate calls. It will be interesting to observe future developments that use different machine learning approaches for the field of SV calling, all of which could potentially improve the field. Here, we propose a few deep learning frameworks illustrating the potential of AI to enhance SV detection that is intended as a discussion point within the community (Fig. 3).

Besides SV detection, machine learning and AI may also be a good option for SV pathogenicity prediction to facilitate clinical application. Although NGS has tremendously improved the resolution of SV detection, it also poses a significant challenge to prioritize and pinpoint a small subset of SV that is clinically relevant. Therefore, accurately discerning the pathogenicity of the SVs identified through NGS testing is profound for its clinical adoption [112]. The guideline for the interpretation and reporting of constitutional CNVs have been jointly proposed by the ACMG and the Clinical Genome Resource (ClinGen), suggesting scoring metrics by integrating reported cases, consistency of phenotype, the pattern of inheritance, and the pathogenic mechanisms of variants to prioritize the CNV pathogenicity [113].

Some efforts have been made for pathogenicity prediction of SV, such as strategies by aggregation of SNP pathogenicity within the SV intervals (e.g., SVscore [114] and AnnotSV [115]) and rule-based approaches for pathogenicity assessment based on the ACMG guideline (e.g., ClassifyCNV [116]). The guideline’s execution relies heavily on domain experts and opinion specific, limiting its application to tackle a large number of CNVs detected by NGS testings [117]. ClassifyCNV is the first tool that automates the implementation of the updated ACMG guidelines to classify CNVs, which is suitable for integration into NGS analysis pipelines. However, current SV pathogenicity scoring tools mainly focus on protein-coding regions, and no single approach integrates the distribution of CNVs across ethnic groups to more precisely predict CNV pathogenicity.

Machine learning-based approaches by integrating different genome features and ethnic information across the population could effectively improve the prediction performance of SV pathogenicity in the whole genome scale. As part of the FDA-led SEQC II effort, we developed a novel machine learning-based framework X-CNV (www.unimd.org/XCNV) by integrating over 30 informative features correlating with SNV pathogenicity, to quantitively predict the pathogenicity of CNVs across various ethnic groups across approximately 93% of the human genome [118]. The proposed X-CNV outperformed all the current CNV pathogenicity tools with an AUC of 0.94. Moreover, we developed a meta-voting prediction (MVP) score to quantitively measure the pathogenic effect aligned with the ACMG guideline to enhance clinical application. In the current version of X-CNV, we employed the XGBoost algorithm, and further investigations on advanced deep learning algorithms may promise enhanced performance.

Although machine learning and AI shed light on SV detection and interpretation, more comprehensive evaluation and further investigation on the context of use are highly recommended towards a robust, secured, privacy-preserving, and explainable machine learning and AI solution for clinical application. The U.S. Food and Drug Administration (FDA) recently issued the “Artificial Intelligence/Machine Learning (AI/ML)-Based Software as a Medical Device (SaMD) Action Plan” to enable the FDA and manufacturers to evaluate and monitor a software product in a lifecycle-based regulatory framework for machine learning and AI technologies and allow for modifications to be made from real-world learning and adaptation (https://www.fda.gov/medical-devices/software-medical-device-samd/artificial-intelligence-and-machine-learning-software-medical-device). Crowdsourcing efforts led by government agencies such as NIST and FDA will be tremendously helpful to prioritize fundamental and translational AI research consistent with regulatory priorities for robust, safe, secure, and privacy-preserving machine learning in different real-world applications. One example is PrecisionFDA, led by the U.S. FDA, which is a secure, collaborative, high-performance computing platform to advance precision medicine, inform regulatory science, and enable improvements in health outcomes. Several PrecisionFDA challenges have been launched, such as calling variants from short and long reads in difficult-to-map regions to standardize NGS testing in the precision medicine practice (https://precision.fda.gov/challenges) [119]. The SEQC-II consortium will release a new challenge on machine learning and AI powering genetic variant identification in the PrecisionFDA to take advantage of crowdsourcing efforts to better understand AI’s pros and cons in the context of NGS testing in a clinical setting.

### Towards clinical implementation of NGS-based SV detection

Although NGS has tremendously improved the resolution of SV detection and facilitated our understanding of disease etiology and pathogenesis, substantial challenges remain for its full clinical implementation [34]. The influential critical aspects of the clinical implementation of NGS-based SV detection are multifactorial, and the significant factors include pathogenicity assessment, reporting, accreditation, analytical, and clinical validation. The current first-line clinically accredited laboratory genetic tests are still array-based technologies such as array comparative genome hybridization (aCGH) or SNP arrays, which are only capable to detect CNV down to a resolution of ~ 50 kb and unable to detect other types of SV events, such as inversions or balanced translocations. NGS has shown high sensitivity, specificity, and reproducibility for the SV less than 50 bp [36]. Besides, NGS shows its promise in the large SV detection, although substantial space for further improvement for enhanced sensitivity and specificity.

The strategies of promoting NGS towards its clinical implementation and adoption have been intensively discussed elsewhere [120, 121]. One of the outstanding prerequisites of NGS application in a clinical setting is whether the NGS-based SV detection is superior to the current first-line clinically accredited genetic testing. Gross et al. [122] conducted a comparative analysis between NGS-based CNV detection and clinically accredited array-based strategy across a clinical cohort of 79 rare and undiagnosed cases. The study showed CNV calls from NGS are at least as sensitive as those from microarrays while only creating a modest increase in the number of variants interpreted (~10 CNVs per case). Encouragingly, 15% of these incidental or secondary findings (ISFs) from NGS could be confirmed with an orthogonal approach.

The potential high false-positive rate of NGS-based SV detection is one of the major concerns for its clinical applications compared to currently clinically accredited laboratory genetic tests. Since the NGS pipeline for SV detection involves multiple steps, a standard framework for managing and standardizing the NGS-based SV detection is urgently needed for clinical application. To fill the gap, a ClinSV was recently proposed to provide a “one-stop” solution for WGS based SV integration, annotation, prioritization, and visualization [123]. The proposed ClinSV achieved a low false rate (1.5~4.5%) and high reproducibility (95~99%), and high sensitivity (99.8%) for simulated pathogenic ClinVar CNVs > 10 kb and 100% from clinically accredited array-based testing. More importantly, ClinSV identified actionable variants in 22 of 485 patients (4.7%), 35~63% of which were not identified by current clinical microarray designs.

#### Future directions

The accurate and reproducible detection of SVs is required for use in clinical applications. As a direct benefit of the rapid evolution of genomic technologies coupled with the work of different stakeholders, an increased appreciation of the contribution of SVs in genetic diversity and disease etiology continues to emerge [4, 124–126]. A strategic and thoughtful application of genomics technologies can drive seamless harmonization of diverse strategies to provide reliable and robust SV detection. Here, we have made several proposals to improve the accuracy and reproducibility of SV detection. Some aspects remain to be addressed such as the reliable estimation for SV pathogenicity [115, 116, 118] and the association between SV and complex trait loci [127]. Furthermore, our perspective is only focused on the human genome; however, SV events are also widely distributed in other species [128, 129]. One notable example is SV events in SARS-CoV-2, which may contribute to COVID-19 transmission and severity [130, 131]. Additionally, we did not discuss RNA-seq based gene fusion detection. Gene capture strategies, genomics technologies selection and calling algorithms divergence have been extensively studied under SEQC II. Specifically, the SEQC Oncopanel working group generated multi-platform and multi-lab long/short-read RNA sequencing data along with conventional genomics technologies such as stranded/polyadenylated/ribosome-depleted sequencing [28, 29]. This enables a comprehensive assessment of gene fusion detection capability using the common reference material UHRR.

The field of SV detection continues to expand with advances in genome technologies, the establishment of and more reference standards and the release of high-quality calling sets. These resources allow us to further investigate some of the critical questions that remain and facilitate novel genomics technology development towards reliable and comprehensive SV identification (see Outstanding Questions). Notably, regulatory attention is on standardizing structural variant detection and on classification for convenient clinical adoption [132]. We suggest establishing a bridge among different stakeholders to establish best-practice recommendations and quality control to encourage the uptake of SV diagnosis into clinical practice. Additionally, we argue that innovations in AI may help in tackling key challenges in SV detection and provide alternative options to develop more accurate and robust approaches. However, these proposed deep learning frameworks still require close examination and feasibility analysis. For example, there are still multiple challenges to fully implement deep learning framework in SV calling such as lack of training data, divergency of performance on complexity of SV and regional differences.

## Conclusions

The ongoing development of many genomics technologies sees a period of excitement, huge investment and perhaps disappointment that, in turn, may trigger a new wave of innovation. We foresee that more accurate and reproducible SV detection approaches will emerge soon, generating a more complete picture of the landscape of the human genome. Next steps will require different stakeholders to work towards a common goal of further resolving the difficulty in SV detection and accelerating clinical application.

## Supplementary Information

**Additional file 1.** Review history.
