# Author Correction: Towards accurate and reliable resolution of structural variants for clinical diagnosis

**DOI:** 10.1186/s13059-022-02773-0

**Published:** 2022-09-20

**Authors:** Zhichao Liu, Ruth Roberts, Timothy R. Mercer, Joshua Xu, Fritz J. Sedlazeck, Weida Tong

**Affiliations:** 1 National Center for Toxicological Research, U.S. Food and Drug Administration, Jeferson, AR 72079 USA; 2 ApconiX, BioHub at Alderley Park, Alderley Edge, SK10 4TG UK; 3 University of Birmingham, Edgbaston, Birmingham, B15 2TT UK; 4 Australian Institute for Bioengineering and Nanotechnology, University of Queensland, Brisbane, QLD Australia; 5 Garvan Institute of Medical Research, Sydney, NSW Australia; 6 St Vincent’s Clinical School, University of New South Wales, Sydney, NSW Australia; 7 Human Genome Sequencing Center, Baylor College of Medicine, One Baylor Plaza, Houston, TX 77030 USA

**Correction: Genome Biol 23, 68 (2022)**

**https://doi.org/10.1186/s13059-022-02636-8**

Following the publication of the original paper [1], the authors reported a few errors. They would like to make the below corrections under the “**Somatic Benchmarking**” section, “**Determination of sequencing technologies to improve SV detection**” section, and **Fig.** **2**.

In “**Somatic Benchmarking**” section,

*“The HCC1395 cell line has been characterized with conventional genomics approaches such as cytogenetic analysis [49] and array-based comparative genomic hybridization [50], consisting of rich genetic variant types including ~40,000 SNVs, ~2000 small indels, CNAs covering over 50% of the genome, more than 250 complex genomic rearrangements [51], and an aneuploid genome and BRCAness [52].”*

To

Supplementing WGS and WES, conventional array-based and cytogenetic-based genomics approaches [49,50] have been employed to characterize the HCC1395 cell line resulting in various genetic variant types, including a substantial number of SNV/small indels and complex SVs identified [35,51,52].

*“Moreover, the HCC1395 DNA was pooled with HCC1395BL DNA at different ratios to create a range of admixtures that mimicked tumor purity levels of 100%, 75%, 50%, 20%, 10%, 5%, and 0%.”*

To

Moreover, a series of admixtures were generated by titrating the HCC1395 and HCC1395BL DNA with different ratios to mimic the tumor purity levels from 0% to 100% [32].

“**Determination of sequencing technologies to improve SV detection**” section

The SEQC-II adopted multi-platform and multi-lab designs for a comprehensive assessment of reproducibility and accuracy of the detection of SVs. The SV working group set out to investigate the reproducibility and variability of SV calls when the sample was sequenced across multiple sequencing instruments or in different laboratories [31, 32]. Interestingly, current approaches produced a level of variability often associated with false negatives (i.e., missed SVs) in SV calls with current methodologies.

To

The SEQC-II adopted multi-platform and multi-lab designs to comprehensively assess the reproducibility and accuracy of detecting SNVs and small indels [31,32]. The sequencing data could be further leveraged for evaluating the reproducibility and variability of SV calling and benchmark SV calling set development.

**Fig. 2:**
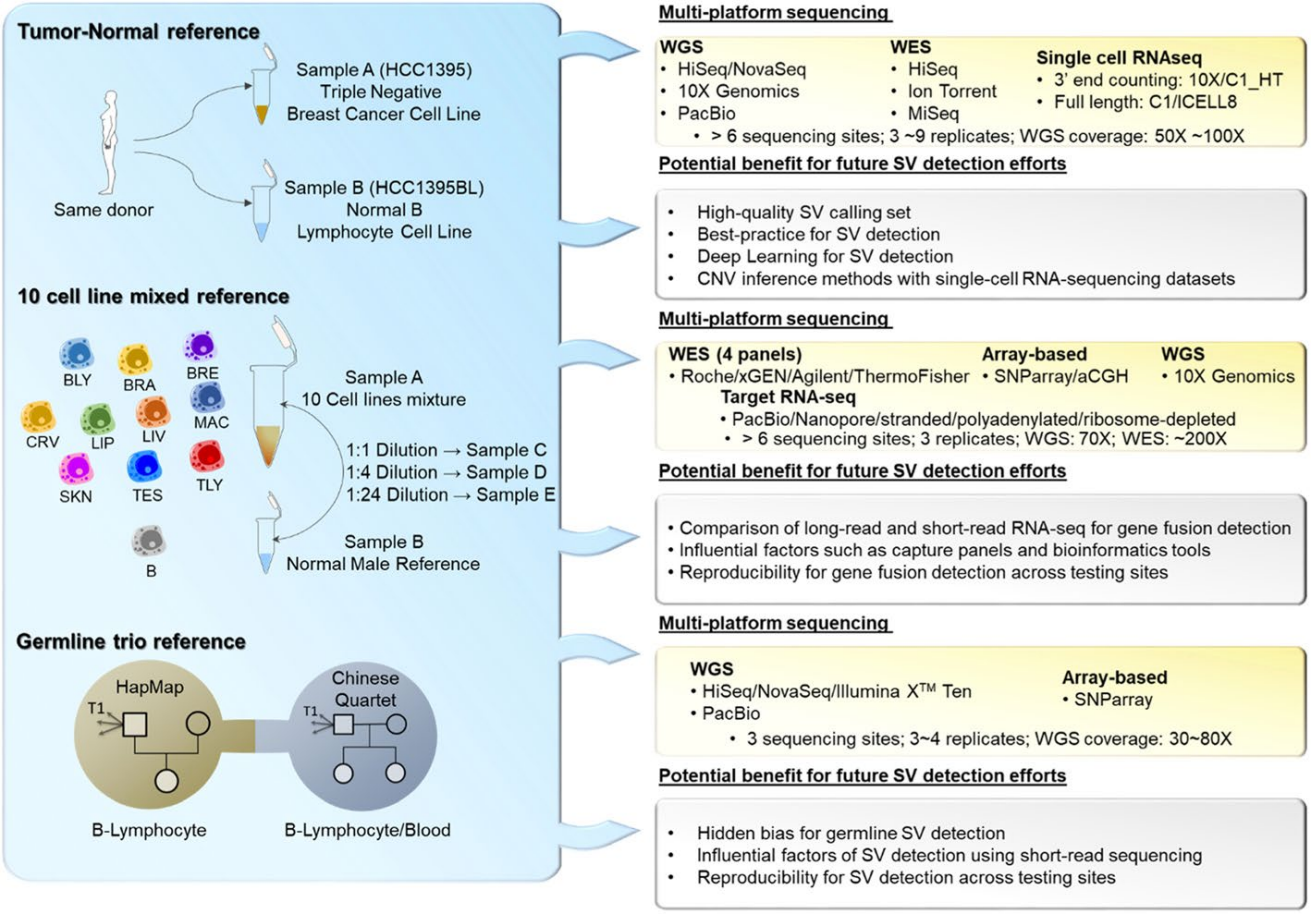
An overview of the reference samples and multiple platform sequencing technologies employed by the SEQC-II consortium, and their potential benefits for future SV detection efforts in the community
