# Supplementary material for: Towards accurate and reliable resolution of structural variants for clinical diagnosis
Source: Genome Biol. 2022 Mar 3;23:68. doi: 10.1186/s13059-022-02636-8 (PMC8892125; doi:10.1186/s13059-022-02636-8)
Supplement: Supplementary file 1 — Additional file 1. Review history. [file 13059_2022_2636_MOESM1_ESM.docx]

Review History

**First round of review**

**Reviewer 1**

**Comments to author:**

This manuscript entitled "Accurate and Reliable Resolution of Structural Variants for clinical diagnosis" reviews available references structural variant (SV) datasets and the approaches used to construct them, the use of specific sequencing technologies in improving SV detection, and discusses the different methods in calling and genotyping SVs, including their advantages and disadvantages. Although it is evident that calling SVs accurately, therefore exploring the necessary steps to reach this purpose, particularly for clinical diagnosis is of high interest, I am not convinced that this article provides insightful discussion, in the methods and ideas it covers, and rather covers said topics superficially, with vaguely formulated points. I would argue that this reduces the usefulness of this review as it does not provide necessary evidence and detail on the pros and cons of each discussed method and technology.

Here are my specific points:

*) In the characterization of standard reference materials section, the authors provides information on the steps taken by the SEQC-II consortium, and shows the usage of tumor-normal cell lines. Although they touch on important matters such as the nature of samples taken, no definitive result or suggestion, or message is provided for the usefulness of what is presented. For example, no specific purpose or insight is given on the advantages of the applied methods by the SEQC-II consortium.

*) Previous work on establishing such truth datasets focused on mixing two normal samples at various percentages to mimic tumor heterogeneity, it would be useful to have detailed reasoning why the proposed approach can be more useful.

*) I find the section from line 24-65 very vague, and not useful due to insufficient reasoning.

*) When discussing the different technologies and what they can deliver, I would find it much more useful if the authors could present general summary statistics from a series of studies in discussing their benefits and drawbacks, alongside reported updated costs in different sequencing use cases.

*) The name of the software is not given in line 8 in page 9.

*) The authors in page 10 compares benchmark datasets, without any summary results, and rather almost intuitively.

*) Similarly, the section "Alignment vs. assembly vs. graph genome approaches" mainly mentions that such approaches exist, without expressing their strengths and drawbacks, or use case suggestions.

*) Page 12, "However unions of all SVs calls across multiple SV calling methods resulted in high false-positive rates...". Although this makes sense and is highly evident, we cannot reach any evidence on this in the manuscript, and this pattern repeats in the rest of the work presented.

*) Similar lack of focus and vagueness is also present in the "Incorporation of machine learning and AI" section.

Citations needed:

Line 38, page 3

line 35, page 11

line 12-14. page 13

**Reviewer 2**

**Comments to author:**

Comments to Author:

In this review article, the authors discuss three aspects that will improve structural variant detection, and ultimately the use of SV analysis in clinical analyses. The authors correctly point out that there are several challenges with developing and benchmarking SV methods, namely a lack of biological standards, lack of high-quality call sets, limitations of available technology. However, all the insights discussed are well established in the literature and reinforced in most genome-wide SV or SV analysis tool, or human long-read publication, limiting the interest, and novel contributions of this manuscript.

Major points

* Beyond the final perspective section, most of the paper was not restricted to clinical applications, instead focussing on SV in general. If this is the be the focus, then I'd recommend more focus on actual clinical uses, in laboratories, with consideration for pathogenicity assessment, reporting, accreditation, analytical and clinical validation. I'd recommend highlighting the performance and findings from recent tools that have been benchmarked for clinical use, on patient materials, such as PMID: 30293986 and PMID: 33632298

* In the section on consensus methods, the authors would benefit from distinguishing between "SV callers" which typically only look at split and spanning reads, or assembly, vs the interrelated "CNV callers" which spot changes in depth of coverage and/or BAF signals; methods that integrate these signals.

* The title would benefit from reframing, for eg "Towards accurate and reliable…"; as it is written, it sounds more like a paper that has developed a method for achieving this.

* The reader is led to expect that Box 2 is about SV detection (see P14, L9 .. to enhance SV detection…), but the section on GAN is about SV simulation. This doesn't flow well. The text in NLP is quite repetitive with the main text (eg sentences on DeepSV).

Minor points

* P5 heterogenicity -> heterogeneity?

* P5 "from the same donor" - as written, this implies that the patient had both B-leukaemia and TNBC

* P5 'the effect of matrix' is an unusual term; do you mean 'the effect of fixatives and sample handling'?

* P6 - complex matrix effects… I still don't understand this term used here.

* P11 - not clear why LOH affects assembly - it should make it cleaner as there's only one haplotype to assemble

* P13, L30 typo DeepVariant

* P14 L8: Need a better linker to box 2, instead of 'Here we propose'. As written, I expected the following text to be these discussion points.

* True set should be truth set, throughout, including Figure 1

* Figure 1 duplication and word wrapping in top right box

* What is Figure 3A, 3B, 3C?

* Box 1 contains placeholders for citations.

* Box 1, Illumine = Illumina

**Authors Response**

**Point-by-point responses to the reviewers’ comments:**

Reviewer #1:

This manuscript entitled "Accurate and Reliable Resolution of Structural Variants for clinical diagnosis" reviews available references structural variant (SV) datasets and the approaches used to construct them, the use of specific sequencing technologies in improving SV detection, and discusses the different methods in calling and genotyping SVs, including their advantages and disadvantages. Although it is evident that calling SVs accurately, therefore exploring the necessary steps to reach this purpose, particularly for clinical diagnosis is of high interest, I am not convinced that this article provides insightful discussion, in the methods and ideas it covers, and rather covers said topics superficially, with vaguely formulated points. I would argue that this reduces the usefulness of this review as it does not provide necessary evidence and detail on the pros and cons of each discussed method and technology.

Here are my specific points:

*) In the characterization of standard reference materials section, the authors provide information on the steps taken by the SEQC-II consortium, and shows the usage of tumor-normal cell lines. Although they touch on important matters such as the nature of samples taken, no definitive result or suggestion, or message is provided for the usefulness of what is presented. For example, no specific purpose or insight is given on the advantages of the applied methods by the SEQC-II consortium.

*) Previous work on establishing such truth datasets focused on mixing two normal samples at various percentages to mimic tumor heterogeneity, it would be useful to have detailed reasoning why the proposed approach can be more useful.

*) I find the section from line 24-65 very vague, and not useful due to insufficient reasoning.

*We appreciate the comments/critiques. We made a major revision on the Characterization of the standard reference materials” section. Specifically, we elaborated on the rationale behind the experimental design (e.g., tumor-normal cell lines) and their usefulness for calibrating the NGS-based SV detection. Please see the Page 5~8.*

*) When discussing the different technologies and what they can deliver, I would find it much more useful if the authors could present general summary statistics from a series of studies in discussing their benefits and drawbacks, alongside reported updated costs in different sequencing use cases.

*Great suggestion. We added Table 1 to compare the popular NGS-based SV detection technologies on pros and cons and cost. Please see the newly added Table 1.*

*) The name of the software is not given in line 8 in page 9.

*Fixed*

*) The authors in page 10 compares benchmark datasets, without any summary results, and rather almost intuitively.

*We added Table 2 to summarize the benchmark generated from SEQC-II efforts. Meanwhile, we added the related description to discuss the previous efforts on benchmark datasets.*

*) Similarly, the section "Alignment vs. assembly vs. graph genome approaches" mainly mentions that such approaches exist, without expressing their strengths and drawbacks, or use case suggestions.

*Thank you so much for the valuable suggestions. We added the strengths and drawbacks of the existing approaches and potential further improvement solutions. Please see pages 14~15.*

*) Page 12, "However unions of all SVs calls across multiple SV calling methods resulted in high false-positive rates...". Although this makes sense and is highly evident, we cannot reach any evidence on this in the manuscript, and this pattern repeats in the rest of the work presented.

*Thank you so much again for the valuable suggestions. We two paragraphs to provide insight on the potential benefit and concerns on meta-callers for SV detection. Please see page 15.*

*) Similar lack of focus and vagueness is also present in the "Incorporation of machine learning and AI" section.

*Thanks a lot for pointing it out. We substantially revised this section. First, we added one part of the potential utility of AI and machine learning on SV pathogenicity prediction. Second, we also discuss the crowdsourcing efforts and regulatory efforts to promote AI and machine learning in the clinical application of NGS-based SV detection. Please see pages 17~19.*

Citations needed:

Line 38, page 3

line 35, page 11

line 12-14. page 13

*Added*

Reviewer #2:

In this review article, the authors discuss three aspects that will improve structural variant detection, and ultimately the use of SV analysis in clinical analyses. The authors correctly point out that there are several challenges with developing and benchmarking SV methods, namely a lack of biological standards, lack of high-quality call sets, limitations of available technology. However, all the insights discussed are well established in the literature and reinforced in most genome-wide SV or SV analysis tool, or human long-read publication, limiting the interest, and novel contributions of this manuscript.

Major points

* Beyond the final perspective section, most of the paper was not restricted to clinical applications, instead focusing on SV in general. If this is the be the focus, then I'd recommend more focus on actual clinical uses, in laboratories, with consideration for pathogenicity assessment, reporting, accreditation, analytical and clinical validation. I'd recommend highlighting the performance and findings from recent tools that have been benchmarked for clinical use, on patient materials, such as PMID: 30293986 and PMID: 33632298

*Great suggestion. We added a new section, “Towards Clinical Implement of NGS-based SV detection,” emphasizing the potential solutions for enhancing the clinical application of NGS-based testings. Particularly, as the reviewer suggested, we highlight the performance and findings from recent tools benchmarked for clinical use on patient materials. Please see Pages 19~20.*

* In the section on consensus methods, the authors would benefit from distinguishing between "SV callers" which typically only look at split and spanning reads, or assembly, vs the interrelated "CNV callers" which spot changes in depth of coverage and/or BAF signals; methods that integrate these signals.

*Thank you so much again for the valuable suggestions. We added wo paragraphs to provide insight on the potential benefit and concerns on meta-callers for SV detection. Please see page 15.*

* The title would benefit from reframing, for e.g., "Towards accurate and reliable…"; as it is written, it sounds more like a paper that has developed a method for achieving this.

*Great suggestion and many thanks. The updated title is “Towards Accurate and Reliable Resolution of Structural Variants for clinical diagnosis”.*

* The reader is led to expect that Box 2 is about SV detection (see P14, L9 .. to enhance SV detection…), but the section on GAN is about SV simulation. This doesn't flow well. The text in NLP is quite repetitive with the main text (eg sentences on DeepSV).

*Thank you so much for the valuable suggestion. We updated Box2, deleted the repetitive descriptions, and changed the contents' presentation order to make it flow better. Moreover, we substantially revised this section. First, we added one part of the potential utility of AI and machine learning on SV pathogenicity prediction. Second, we also discuss the crowdsourcing efforts and regulatory efforts to promote AI and machine learning in the clinical application of NGS-based SV detection. Please see pages 17~19.*

Minor points

* P5 heterogenicity -> heterogeneity?

*Fixed*

* P5 "from the same donor" - as written, this implies that the patient had both B-leukaemia and TNBC

*Fixed*

* P5 'the effect of matrix' is an unusual term; do you mean 'the effect of fixatives and sample handling'?

*Fixed.*

* P6 - complex matrix effects… I still don't understand this term used here.

*Fixed*

* P11 - not clear why LOH affects assembly - it should make it cleaner as there's only one haplotype to assemble

*Fixed*

* P13, L30 typo DeepVariant

*Fixed*

* P14 L8: Need a better linker to box 2, instead of 'Here we propose'. As written, I expected the following text to be these discussion points.

*Thank you so much for the valuable suggestion. We updated Box2, deleted the repetitive descriptions, and changed the contents' presentation order to make it flow better.*

* True set should be truth set, throughout, including Figure 1

*Fixed*

* Figure 1 duplication and word wrapping in top right box

*Fixed*

* What is Figure 3A, 3B, 3C?

*Fixed*

* Box 1 contains placeholders for citations.

*The refs were added.*

* Box 1, Illumine = Illumina

*Fixed*

**Second round of review**

**Reviewer 1**

Although the authors added extra explanations to the specific points/concerns pointed by both reviewers, I think the general lack of clarity in the manuscript remains. I find the organization of the text confusing, and the additional text that is written after the review to be of similar nature. As a result, I find it difficult to follow the manuscript, and find it challenging to accurately assess the novel contributions of this manuscript.

While additional information is provided, I do not think it necessarily responds to the previous review. It is still difficult to clearly discern from the text what aims (e.g. germline SVs, somatic SVs on tumor samples, and what type of SVs (insertions, deletions, or SVs emerging from repetitive vs. unique genomic locations), ... etc. ) are already achieved relatively well, (using which technology/data and set of methodologies), alongside what aims are lacking still (and where they are lacking) and which technology/data and method sets are promising in filling such gaps in using SV analysis for clinical diagnosis, alongside sufficient evidence, e.g. the false positive and false negative call rates or other informative statistics of presented technologies and methods.

In this light, I would suggest reorganization in all sections and narrowing down the focus when necessary.

Regarding the newly added Table 1, although is a good summary on the it still provides no summary statistics on the identification capacity of specific sequencing technologies. What is presented here is well established in the literature.

I find the newly added Table 2 quite useful.

**Reviewer 2**

This revised manuscript is much improved. Changing the title, the addition of the new section on clinical implementation, and the new Figure 2 has helped match the scope of the manuscript better. Most of the suggestions below are minor and editorial in nature.

P5-L15: rewrite ‘Thus challenges, such as cancer or other pathogenic mutations, are unlikely to occur. ‘ P5-L20: these KRAS SNV standards distract from the discussion about SV standards

P5-L19-30: several concepts are rapidly introduced here making this chaotic. Recommend this whole section (indeed pages 5-8) be reformatted for readability, for example starting with general challenges for genome-wide standards, then moving into subsections on germline benchmarking, and somatic benchmarking

P14-L4-L5 Nevertheless…: this sentence is out of place P14-L7: rephrase ‘Up so far’ and ‘the somehow downside’

P14-L11: what is the biggest issue and why are translocations specifically mentioned? Small DUPs and insertions seem quite difficult issues today.

P14-L13-L15: the addition of Figure 2 is welcomed.

P16-L16: rephrase ‘large often CNV’

P16-L17 – bi-allelic do you mean B-allele frequency (using SNP not SNV)

P20-L2 – typo implementation P20-L11: this seems unlikely; citation needed for reproducibility >90% for SV < 50bp

P31: Table 1 is a useful addition to the manuscript. rephrase the limitation, as short-read NGS performs well for >1kb regions. It struggles with shorter CNV detection 50-500bp, and in complex genome regions; 10x: rephrase “currently discontinued”; ONT: specify which technology gives 15Gb/day as it changes so often; HiC: typo ‘products1’

P32 Table 2: PRJNA504037, …/fda_scRNA-seq, the capsule all refer to single-cell RNAseq data; why are they being reported here for somatic CNV analysis? https://biosino.org/node/project/detail/OEP001896 does not work

P40 Figure 3: include sub-labels A-C to match the in text citations

**Authors Response**

**Point-by-point responses to the reviewers’ comments:**

Comments from Reviewer 2

This revised manuscript is much improved. Changing the title, the addition of the new section on clinical implementation, and the new Figure 2 has helped match the scope of the manuscript better. Most of the suggestions below are minor and editorial in nature.

*Thank you so much for your kind word and encouragement.*

P5-L15: rewrite ‘Thus challenges, such as cancer or other pathogenic mutations, are unlikely to occur.’

*Thanks a lot for pointing it out. We deleted this confused sentence.*

P5-L20: these KRAS SNV standards distract from the discussion about SV standards.

*Fully agree. We excluded these irrelevant guidances for clarification.*

P5-L19-30: several concepts are rapidly introduced here making this chaotic. Recommend this whole section (indeed pages 5-8) be reformatted for readability, for example starting with general challenges for genome-wide standards, then moving into subsections on germline benchmarking, and somatic benchmarking

*Fantastic idea. We reorganized this part based on reviewer’s great suggestion. Specifically, we rearranged the contents under three subsections including general challenges for genome-wide reference standards, Germline benchmarking efforts, and somatic benchmarking efforts to increase the readability.*

P14-L4-L5 Nevertheless…: this sentence is out of place

*We deleted this sentence for the clarification.*

P14-L7: rephrase ‘Up so far’ and ‘the somehow downside’

*Fixed*

P14-L11: what is the biggest issue and why are translocations specifically mentioned? Small DUPs and insertions seem quite difficult issues today.

*Thanks a lot for pointing out. we added two sentences and ref to clarify why translocation is the biggest issue.*

*“Short read SV call sets often report in the access of up to 4000 translocations of which ~50-70% are repeat extensions[73]. In contrast most methods can detect 50-300bp insertions these days (e.g., delly and manta), but fail at larger size ranges.”*

P14-L13-L15: the addition of Figure 2 is welcomed.

*We added one sentence to elaborate on the message of the Figure 2.*

P16-L16: rephrase ‘large often CNV’

*Fixed.*

P16-L17 – bi-allelic do you mean B-allele frequency (using SNP not SNV)

*Thank you so much for pointing it out. We fixed based on your great suggestion.*

P20-L2 – typo implementation

*Fixed*.

P20-L11: this seems unlikely; citation needed for reproducibility >90% for SV < 50bp

*Thanks a lot for pointing it out. We rewrote the sentence and added the related references.*

*“NGS has shown high sensitivity, specificity, and reproducibility for the SV less than 50 bp [38]”*

P31: Table 1 is a useful addition to the manuscript. rephrase the limitation, as short-read NGS performs well for >1kb regions. It struggles with shorter CNV detection 50-500bp, and in complex genome regions; 10x: rephrase “currently discontinued”; ONT: specify which technology gives 15Gb/day as it changes so often; HiC: typo ‘products1’

*Thank you so much for your suggestion. We updated the Table 1 based on your valuable suggestions.*

P32 Table 2: PRJNA504037, …/fda_scRNA-seq, the capsule all refers to single-cell RNAseq data; why are they being reported here for somatic CNV analysis? https://biosino.org/node/project/detail/OEP001896 does not work

*They used the same reference materials from somatic mutation working group #1.*

*We fixed the link.*

P40 Figure 3: include sub-labels A-C to match the in text citations

*We updated the Figure 3 with sub labels A-C added.*
